# Nitrogen-Deprivation Elevates Lipid Levels in *Symbiodinium* spp. by Lipid Droplet Accumulation: Morphological and Compositional Analyses

**DOI:** 10.1371/journal.pone.0087416

**Published:** 2014-01-27

**Authors:** Pei-Luen Jiang, Buntora Pasaribu, Chii-Shiarng Chen

**Affiliations:** 1 Graduate Institute of Marine Biotechnology, National Dong-Hwa University, Pingtung, Taiwan; 2 Taiwan Coral Research Center, National Museum of Marine Biology and Aquarium, Pingtung, Taiwan; 3 Graduate Institute of Biotechnology, National Chung-Hsing University, Taichung, Taiwan; 4 Department of Marine Biotechnology and Resources, National Sun Yat-Sen University, Kaohsiung, Taiwan; Louisiana State University Health Sciences Center, United States of America

## Abstract

Stable cnidarian-dinoflagellate (genus *Symbiodinium*) endosymbioses depend on the regulation of nutrient transport between *Symbiodinium* populations and their hosts. It has been previously shown that the host cytosol is a nitrogen-deficient environment for the intracellular *Symbiodinium* and may act to limit growth rates of symbionts during the symbiotic association. This study aimed to investigate the cell proliferation, as well as ultrastructural and lipid compositional changes, in free-living *Symbiodinium* spp. (clade B) upon nitrogen (N)-deprivation. The cell proliferation of the N-deprived cells decreased significantly. Furthermore, staining with a fluorescent probe, boron dipyrromethane 493/503 (BODIPY 493/503), indicated that lipid contents progressively accumulated in the N-deprived cells. Lipid analyses further showed that both triacylglycerol (TAG) and cholesterol ester (CE) were drastically enriched, with polyunsaturated fatty acids (PUFA; i.e., docosahexaenoic acid, heneicosapentaenoic acid, and oleic acid) became more abundant. Ultrastructural examinations showed that the increase in concentration of these lipid species was due to the accumulation of lipid droplets (LDs), a cellular feature that have previously shown to be pivotal in the maintenance of intact endosymbioses. Integrity of these stable LDs was maintained via electronegative repulsion and steric hindrance possibly provided by their surface proteins. Proteomic analyses of these LDs identified proteins putatively involved in lipid metabolism, signaling, stress response and energy metabolism. These results suggest that LDs production may be an adaptive response that enables *Symbiodinium* to maintain sufficient cellular energy stores for survival under the N-deprived conditions in the host cytoplasm.

## Introduction

*Symbiodinium* spp., a unicellular dinoflagellate, is commonly found in mutualistic associations with invertebrates such as corals and anemones, and able to transfer more than 90% of its photosynthetically fixed carbon to the host cytoplasm in which it resides [1]. This intracellular symbiosis (i.e. the endosymbiosis) has been the focus of intensive research due to its pivotal role in maintaining the health of corals and homeostasis of the marine ecosystem.

Numerous studies have highlighted the crucial role of nutritional status and nutrient transfer in these endosymbioses [2]. The supply of organic carbon by *Symbiodinium* to the hosts and the recycling of essential nutrients during such associations have contributed to the success of coral reefs in nutrient-limited tropical seas [3]. On the other hand, the growth of *Symbiodinium* is dependent on nutrients from various sources including exogenous seawater, host catabolism, and host heterotrophy [4], [5]. Nitrogen, which is one of the most important essential nutrients, can be excreted as ammonium by the host [6].

The host metabolism plays a significant role in regulating the nutritional status of its endosymbionts (i.e. the symbiotic *Symbiodinium*) [7]. For instance, it has been shown that the endosymbionts were present in nutrient-limited environments [8], [9], particularly with respect to nitrogen sources. Using the infrared microspectroscopy, it has been shown that the concentration of nitrogenous compounds was significantly lower in endosymbionts, compared to that of free-living *Symbiodinium* [9]. It suggests that the cytoplasm of the host cell may be nitrogen-deficient and alter various physiologies of the endosymbiont. In examining the effect of nutrient supplement of host anemones on their endosymbionts, Zhu and colleagues have observed that there was an increase formation of lipid droplets (LDs) in endosymbionts after 45 days of nutrient starvation on host sea anemones [10]. However, the cellular and molecular mechanisms of this regulation remain to be elucidated.

Nitrogen deprivation represents an important source of stress for microalgae, which causes various changes in cellular metabolism and development. For example, treatments with nitrogen deprivation tend to elevate lipid production in algae [11], [12], such as increases of cellular triacylglycerols (TAGs) stored in the cytoplasmic lipid droplets (LDs) [13]. TAGs, which are composed primarily of saturated and monounsaturated fatty acids, can be efficiently packed into the cell and generate more energy than carbohydrates upon oxidation, thus constituting the best reserve for rebuilding the cell upon returning to homeostatic conditions [14]. The proteins associated with these LDs have been the focus of investigations in order to elucidate the mechanism of LD formation [15]. Recent studies have shown that the LD-associated protein in *Chlorella* cells belong to the caleosin family [16], [17]. Another lipid droplet protein, the major lipid droplet protein (MLDP), was also revealed in the LDs of various green microalga including *Chlamydomonas reinhardtii*, *Haematococcus pluvialis* [18], [19] and *Dunaliella* [20].

In order to elucidate the mechanism that allows the endosymbionts to adapt to the nitrogen-limited environment of cnidarian hosts, the present study aims to first examine the cellular response of free-living *Symbiodinium* (clade B) to nitrogen deprivation treatment. To the best of our knowledge, the effect of nitrogen deprivation in free-living *Symbiodinium* spp. has never been described, mainly due to the relative difficulty of cultivating *Symbiodinium* spp. with synthetic culture media [21]. This is an unfortunate knowledge dearth, as nitrogen is an important nutrient required for the metabolism of *Symbiodinium* [22]. Under nitrogen deprivation, the specific goal here was to examine the changes in cellular biology, including the proliferation, lipid contents and ultrastructure in free-living *Symbiodinium* spp. Results show that there is increased formation of LDs, a phenomenon similar to that occur in symbiotic *Symbiodinium*. Subsequently, LDs of these N-deprived cells were purified for biochemical analyses for proteins and lipids. Results of the present study should provide important information in elucidating possible regulatory mechanisms underlying LDs formation in nitrogen-deficient environments such as those in the symbiotic association with Cnidaria.

## Materials and Methods

### *Symbiodinium* culture and the nitrogen-deprivation treatment

The free-living *Symbiodinium* spp. (clade B) used in this study were originally isolated from the sea anemone *Aiptasia pulchella* [9]. They were cultured in the f/2 medium [23] in filtered seawater (FSW) at room temperature under a photosynthetically active radiation (PAR) of 40 µmol m^−2^s^−1^ in a 12-h light/12-h dark (12L/12D) cycle. Media were prepared with two different concentrations of nitrogen (as NaNO_3_). Two new batch cultures were grown, one in nitrogen-deficient f/2 medium (no NaNO_3_ was added to the medium) and the other in nitrogen-sufficient f/2 medium (0.882 mM NaNO_3_).

### *Symbiodinium* clade identification

The genetic identity (18S rDNA) of the cultured *Symbiodinium* was examined by PCR-RFLP (Polymerase chin reaction-Restriction fragment length polymorphism) analysis [24], and shown to be from clade B. *Symbiodinium* DNA was extracted using a plant genomic DNA extraction miniprep system (VIOGENE, Taipei). Basically, *Symbiodinium* nuclear small subunit (n18S-rDNA) was amplified by PCR) from 3 replicate extracts of each of the two cultures using the primers, ss5z (an equimolar mixture of the oligonucleotides 5′-GCAGTTATAATTTATTTGATGGTCACTGCTAC-3′ and 5′-GCAGTTATAGTTTATTTGATGGTTGCTGCTAC-3′) and ss3z (5′-AGCACTGCGTCAGTCCGAATAATTCACCGG-3′) and digested with the restriction enzyme, *Taq* I and *Sau*3A I (Promega, USA). Digestion products were separated by electrophoresis on 1.5% 0.5x TAE (Amresco, USA) agarose gels, to generate the RFLP pattern. RFLP pattern analysis was compared to the literature [24] to assign each culture to one of the established *Symbiodinium* n18S-rDNA RFLP clades.

### Cell density analysis

*Symbiodinium* proliferation was examined with hemocytometer-based cell counting. Cell densities were determined daily by placing an aliquot of well-mixed culture suspension on a Neubauer hemocytometer (Marienfel, Germany) under a Axioskop 2 Plus microscope (Zeiss, Germany) connected to a CCD (charge-coupled device) camera (Photometrics. USA)

### Chlorophyll a and protein determinations

*Symbiodinium* were harvested by centrifugation at 12000 g for 10 min from the control and nitrogen-deprived culture. For each replicate during the analysis, a volume of culture containing 5×10^5^ cells was used. Chlorophyll *a* was extracted in 90% acetone (v/v), and their amount was estimated spectrophotometrically as previously described [25]. The protein content was determined using the BCA protein assay kit (Invitrogen, USA) on the same samples used for chlorophyll quantification.

### The isolation of lipid droplets (LDs) from nitrogen-deprived *Symbiodinium*

LDs were isolated from the nitrogen-deprived culture (1L) on days 5 and 7 by first homogenizing at 4°C in a “grinding buffer” (0.6 M sucrose in 10 mM sodium phosphate buffer, pH 7.5) according to a published procedure [26]. After filtration, each 8-ml portion of the homogenate was placed at the bottom of a 12-ml centrifuge tube, and 2-ml of “flotation buffer” (0.4 M sucrose in 10 mM sodium phosphate buffer, pH 7.5) was layered on top. The tube was centrifuged at 35,000×g for 60 min in a swinging-bucket rotor centrifuge (Beckman Coulter, USA). LDs on the top layer were collected and re-suspended in the “detergent washing solution” (0.4 M sucrose, 0.1% Triton X-100 in 10 mM sodium phosphate buffer, pH 7.5) to remove non-specifically associated proteins. After a further centrifugation, LDs on the top were collected and then re-suspended in the floating buffer to remove excess detergent. The washing step was repeated two more times. Finally, the purified LDs were re-suspended in the grinding buffer and stored at −20°C until further analysis.

### Structural integrity of *Symbiodinium* LDs

The structural integrity of LDs isolated from *Symbiodinium* was assessed by examining the surface properties (steric hindrance and electrostatic repulsion) that accounted for the aggregation of LDs without fusion at pH 6.5 [26]. *Symbiodinium* LDs suspended in 5 mM sodium phosphate buffer, pH 7.5 or 6.5, were kept at 23°C for 6 hrs. To confirm that the steric hindrance was provided by surface proteins, a 2-ml preparation of *Symbiodinium* LDs was subjected to trypsin (2.5 µg; bovine pancreas type III, Sigma, USA) digestion at 37°C for 30 min.

### Lipid analyses

All solvents for lipid analyses were analytic grade. Lipid contents of 3 replicates from the nitrogen-sufficient f/2 medium and nitrogen-deprived culture *Symbiodinium* cells were extracted by the Bligh and Dyer procedure [27]. Neutral lipids in the LDs isolated from *Symbiodinium* cells were extracted with 150 µl of chloroform/methanol (2∶1, v/v). After centrifugation, the lower chloroform fraction was collected for the analysis by thin layer chromatography (TLC) (Analtech, USA) with the solvent system modified from previous reports [28], [29]. Briefly, TLC was first developed to the R_f_ = 1 position in hexane. The plate was air-dried and then developed to the top (R_f_ = 1) in benzene. The plate was air-dried and then developed to the top (R_f_ = 0.5) in hexane: diethyl ether: acetic acid (70∶30∶1 v/v/v). The lipid visualization on TLC plates was performed by staining with 0.03% Coomassie blue R 250 (Sigma, USA) dissolved in 20% methanol containing 0.5% acetic acid [30]. Concentrations of individual lipid species were then quantified using the Metamorph Image Processing system (Molecular Devices Inc., Toronto, Canada) based on calibration curves of individual lipid standards (Wax ester: Sigma-Aldrich, USA; TAGs [mixed triacylglycerides: tricaprin, tricaprylin, trilaurin, trimyristin, tripalmitin]: Sigma-Aldrich, USA; Cholesterol: Avanti Polar Lipids, USA; CE: Sigma-Aldrich, USA) co-run on the same TLC plate. To analyze the phospholipids in extracted lipids, chloroform: acetic acid: methanol: water (70∶25∶5∶2, v/v/v/v) was used as the TLC solvent system.

### Analyses of fatty acid composition in cellular TAGs by gas chromatography (GC)-mass spectrometry (MS)

*Symbiodinium* cells collected after five days of culture in either nitrogen-enriched (control) or nitrogen-deprivation f/2 medium were dried by lyophilization. Total lipids in dried cells were extracted and separated by TLC as previously described. TAGs on TLC plates was first identified by 0.001% primuline spraying (in 80% acetone), and then extracted by the Bligh and Dyer procedure [27]. Isolated TAGs were first saponified in 1N sodium hydroxide-methanol solution for 15 min at 80°C. The fatty acids were esterified in 14% boron trifluoride-methanol solution for 15 min at 100°C. After hexane extraction, the fatty acid methyl esters were analyzed on a gas chromatograph (GC, Varian CP-3800) and a mass spectrometer (Varian 320 MS) operated in full scan mode; scan range from 100 to 450 m/z. The column was a CP-Sil88 capillary column of length 20 m, 0.25 mm i.d., and the stationary phase had a film thickness of 0.2 µm. Helium was used as the carrier gas at a flow rate of 0.8 ml min^-1^. The temperature program was as follows: held at 50°C for 1 min, 50–200°C with 8°C min^−1^, held for 5 min, from 200–230°C with 20°C min^−1^. Retention times and mass spectra were compared against the NIST02 library (National Institute of Standards and Technology, Gaithersburg, MD, USA) to identify fatty acids. Saturn GC/MS Workstation v6.9.3 software (Varian) was used to visualize spectra, integrate areas under peaks and search the library. Peaks of fatty acids were identified, and the relative amount of individual fatty acid was calculated by the integrated area percentage among total fatty acids.

### Fluorescent microscopy

BODIPY 493/503 (4,4-difluoro-1,3,5,7-tetramethyl-4-bora-3a,4a- diaza-s-indacene) is a fluorescent lipophilic stain [31] widely used to label lipid droplets in plants. *Symbiodinium* and purified LDs were stained with 38.2 µM BODIPY 493/503(Invitrogen, USA) in the dark for 20 min at RT. The stained cells and LDs were visualized using the fluorescence microscope (Zeiss, Germany).

### The transmission electron microscopy and imaging analysis

To investigate the intracellular accumulation of LDs, *Symbiodinium* cells under nitrogen-deprivation treatment were collected and fixed in 2.5% glutaraldehyde and 2% paraformaldehyde in 100 mM sodium phosphate containing and 5% sucrose (pH 7.3) for 2.5 h at 4°C. They were then rinsed with 100 mM sodium phosphate buffer at 4°C. Cells were then post-fixed in 1% OsO_4_ in 50 mM sodium phosphate (pH 7.3) for 1 hr at 4°C. The cell aliquots were then washed three times for 15 min each with the same buffer and dehydrated by a graded ethanol series (50, 70, 80, 90, 95 and 100%) before embedding in LR white Resin. Thin sections (70 nm) cut by a Leica Reichert Ultracut R were collected on nickel grids, post-stained with 2.5% uranyl acetate and 0.4% lead citrate, rinsed 3 times with water, and the samples were viewed on a JEM-1400 transmission electron microscope (JEOL, Japan). In order to determine the LDs area from the acquired images, the ratio of the actual length to pixel was first determined by distance calibration using the scale bar of the acquired transmission electron microscopy (TEM) image. Individual LDs were selected by threshold adjustment, and the area (µm^2^) of individual LDs was calculated with Metamorph's region measurement function.

### SDS-PAGE and Western blotting

Proteins from *Symbiodinium* cells and LDs were extracted with an equal volume of 2x sample buffer according to the suggestions in the Bio-Rad (Bio-Rad, USA) Trans-Blot instruction manual and resolved by SDS-PAGE using 15% (w/v) polyacrylamide in the separating gel and 4.75% polyacrylamide in the stacking gel [32]. After electrophoresis, the gel was stained with Coomassie Blue R-250 and then destained with methanol/acetic acid. For Western blotting, proteins were transferred from SDS-PAGE onto a nitrocellulose membrane in a Trans-Blot system (Bio-Rad, USA) according to the manufacturer's instructions. The membrane was subjected to immune-detection using a rabbit anti-ribulose-1,5-bisphosphate carboxylase/oxygenase (Rubisco) large subunit (1;2000 dilution; Cat. AS0037, Agrisera, Vannas, Sweden). After washing, the membrane was incubated with secondary antibodies conjugated with goat anti-rabbit horseradish peroxidase (HRP). The membrane was subsequently washed and resulting proteins visualized using SuperSignal West Pico Chemiluminescent substrate kits (Thermo Fisher Scientific, USA) according to the manufacturer's recommendations and visualized on a Brand Vilber Lourmat Model Fusion FX7 gel-doc under the chemiluminescent settings for 2 min.

### In-gel digestion of the lipid droplet proteins in *Symbiodinium* spp

Five protein bands of *Symbiodinium* lipid droplet resolved by SDS-PAGE were manually excised from the gel and ground into pieces. After washing with 50% acetonitrile and 50% acetonitrile/25 mM ammonium bicarbonate, the protein was reduced and alkylated at 56°C for 45 min in 10 mM dithiothreitol and 55 mM iodoacetamide in 25 mM ammonium bicarbonate, followed by overnight in-gel digestion with 0.1 µg in 15 µl of TPCK-treated modified porcine trypsin (Promega, USA) in the same buffer at 37°C. The supernatant containing tryptic peptides was combined with two more extracts of the gel by 50% acetonitrile/5% formic acid. The sample was analyzed by matrix-assisted laser desorption/ionization-mass spectrometry (MALDI-MS) and MALDI-MS/MS. All data were acquired by quadrupole-time-of-flight (Q-TOF) hybrid mass spectrometers (Micromass Q-Tof Ultima, Manchester, UK, and Applied Biosystems QSTAR, USA), in which α-cyano-4-hydroxycinnamic acid was used as the matrix. The low-energy collision-induced dissociation MS/MS product ion spectra acquired from Q-TOF Ultima and QSTAR were analyzed by Micromass ProteinLynx™ Global Server 2.0 and Applied Biosystems BioAnalyst™ data processing software, respectively. For protein identification, the acquired MS/MS spectra were automatically searched against the NCBInr database using the Mascot search program (www.matrixscience.com) restricted to all entries taxonomy. The mass tolerance parameter was 20 ppm, the MS/MS ion mass tolerance was 1 Da, and up to one missed cleavage was allowed. Variable modifications considered were methionine oxidation and cysteine carboxyamidomethylation. Positive identification of proteins was confirmed by observation of at least one of the following criteria: (i) the total number of matched peptides (mps) is more than 2, or (ii) the mps equals 2 with two different matched peptides, or (iii) the MOWSE score has to be higher than 70 which indicates identity or extensive homology (p<0.05).

### Statistical analysis

All statistical analyses were performed using SigmaStat 3.5 (Systat software, Chicago, IL, USA). The results were expressed as mean±SD (standard deviation of the mean).

## Results

### Nitrogen-deprivation induces lipid content increase in *Symbiodinium*

**Figure 1:**
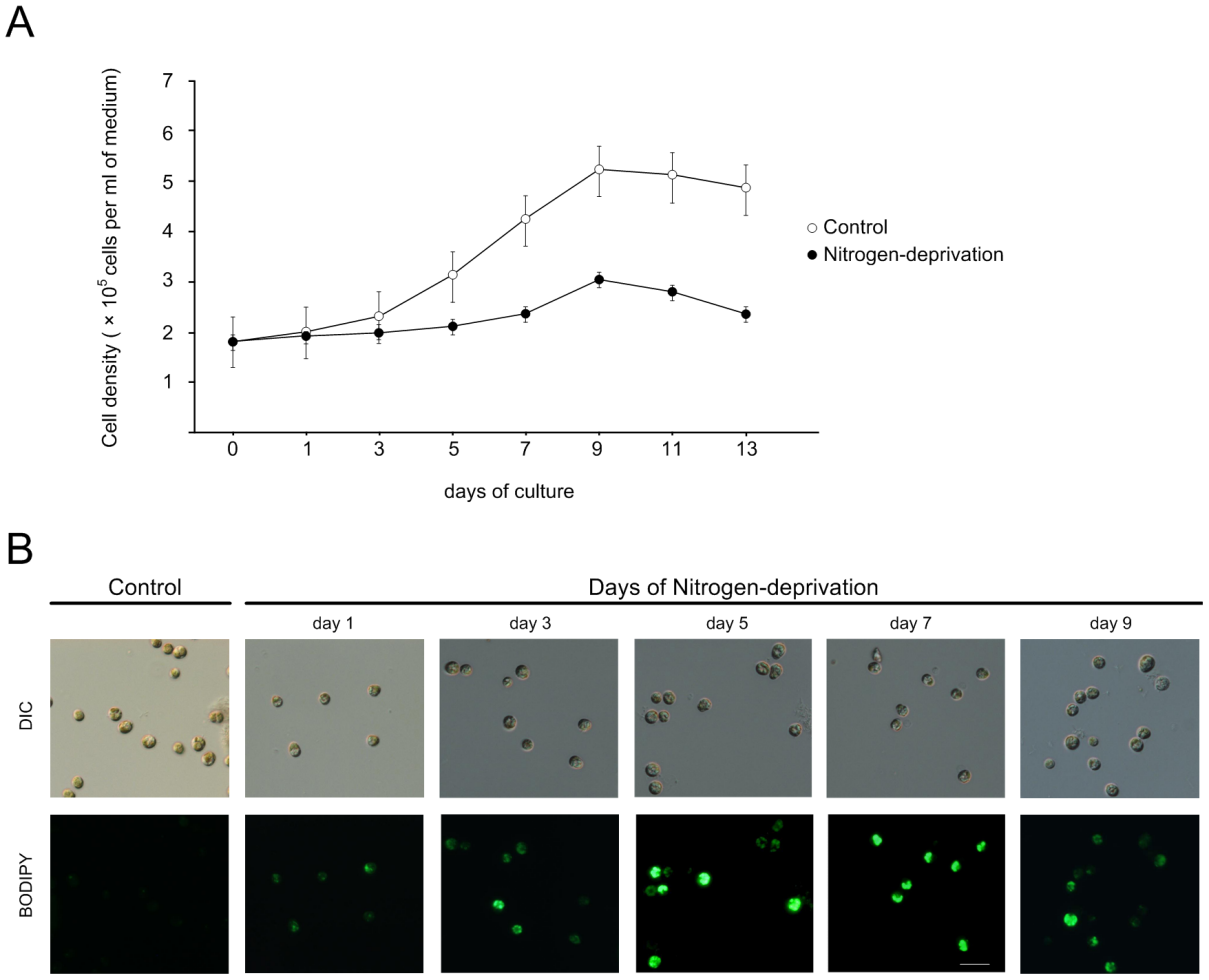
Effect of nitrogen-deprivation on the cell proliferation and lipid accumulation in *Symbiodinium*. (A) Growth of *Symbiodinium* cells cultivated in control versus nitrogen-deprivation media. The data represents mean ± SD (n = 3). (B) The visualization of neutral lipid accumulation using BODIPY 493/503 in control vs. nitrogen-deprived cultures. Scale bar, 10 µm.

To examine the effects of nitrogen deprivation on *Symbiodinium* growth and lipid accumulation, free-living *Symbiodinium* in f/2 medium at the early stationary phase were transferred to nitrogen-free f/2 medium. As shown, cells cultivated in the nitrogen-deprived medium proliferated more slowly than those cultivated in the normal medium, particularly after three days of experimentation (Fig. 1A).

The lipid accumulation in nitrogen-deprived *Symbiodinium* could be visualized by staining the cells with a neutral lipid specific dye BODIPY 493/503. In *Symbiodinium* grown in control medium, little lipid accumulation was observed as shown by very dim BODIPY staining (Fig. 1B). On the contrary, BODIPY fluorescence gradually increased in cells with the nitrogen-deprivation treatment (Fig. 1B; day 1 to day 9), indicating an increase of lipid accumulation.

**Figure 2:**
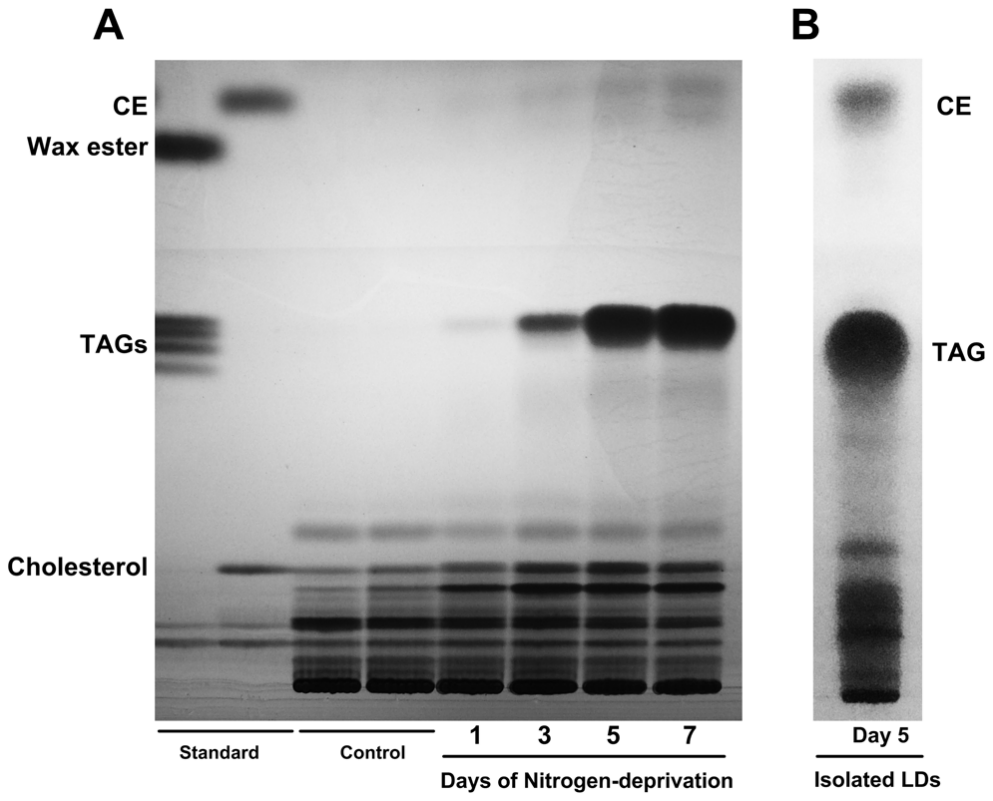
The TLC analysis of lipids extracted from *Symbiodinium* spp. cells. The comparison between control and nitrogen-deprivation treated cells (A). The lipid content of the purified LDs from *Symbiodinium* after five days of nitrogen deprivation is shown in (B).

The change in lipid content during nitrogen-deprivation treatment was then analyzed by TLC (Fig. 2). In the nitrogen-deprivation culture, *Symbiodinium* began to accumulate neutral lipids including TAGs and CEs (cholesterol esters); both of which were very low (<1 pg per cell) in control culture (Fig. 2A). Concentration of both TAGs and CEs increased to reach maximal levels (168.57±4.93 and 13.5±0.52 pg cell^−1^, respectively) after seven days of nitrogen-deprivation treatment (Table 1).

**Table 1:** TAGs and CEs accumulation in *Symbiodinium* spp.

Lipid concentrations (pg/cell)	Control	Days of nitrogen-deprivation			
		Day 1	Day 3	Day 5	Day 7
TAGs	tr	1.90±0.51^ab^	96.55±0.71^c^	163.50±5.54^d^	168.57±4.93^de^
CEs	tr	tr	2.73±0.93^a^	8.95±0.49^b^	13.50±0.52^c^

Lipid contents of *Symbiodinium* in the control and nitrogen-deprivation treatments were analyzed by TLC. Data are presented as mean±SD (N = 3). Superscript a-d denote statistical significance within control and nitrogen-starvation treatments (P<0.001). tr, trace (<1 pg).

**Figure 3:**
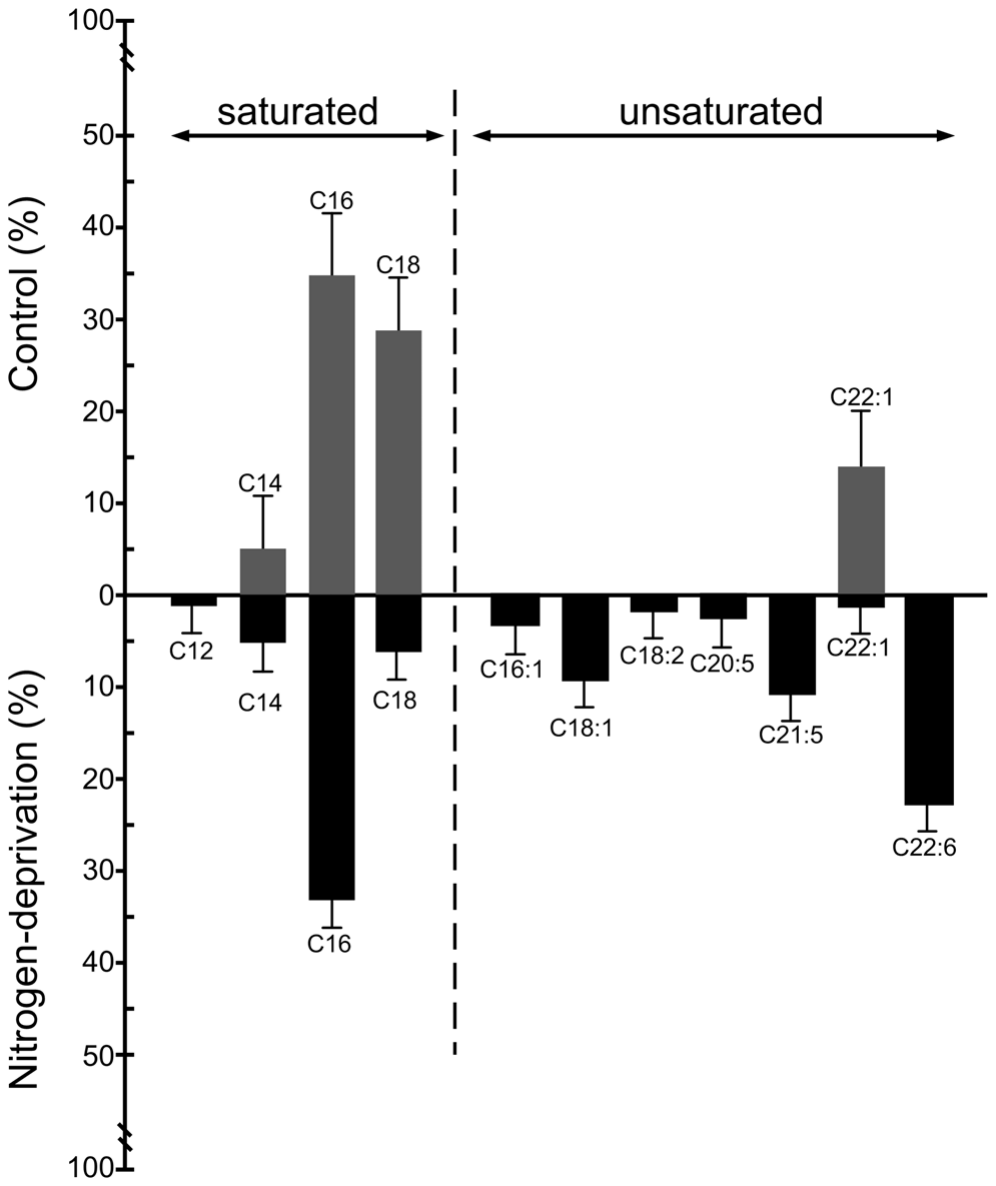
The change of fatty acid compositions in TAGs of *Symbiodinium* after five days of nitrogen deprivation. Relative amounts (%) of fatty acid compositions in purified TAGs from total *Symbiodinium* were determined (see the “Materials and methods” section). The data represents mean ± SD (n = 3).

As the amount of TAG was significantly increased by nitrogen-deprivation treatment, their fatty acid compositions in control and nitrogen-deprivation treatment for five days were further analyzed by GC-MS (Fig.3). In the control group of *Symbiodinium*, four major fatty acids were identified, such as C14:0, C16:0, C18:0 and C22:1. Among them, C16:0 and C18:0 were the most abundant, occupying approximately 38% and 32% of total fatty acids, respectively. After cultivation in nitrogen-deprived medium for 5 days, the percentages of C14:0 and C16:0 remained unchanged. Nevertheless, C18:0 drastically decreased from about 32% to 6.4%. On the other hand, apart from the C12:0 and C22:0, the amount of numerous polyunsaturated fatty acids (PUFAs) such as C16:1, C18:1, C18:2, C20:5, C21:5 and C22:6, increased significantly. In other words, the proportion of PUFAs over saturated fatty acids (SFA) in TAGs increased in *Symbiodinium* cells under the nitrogen-deprived condition.

### Ultrastructural changes in *Symbiodinium* by nitrogen-deprivation

**Figure 4:**
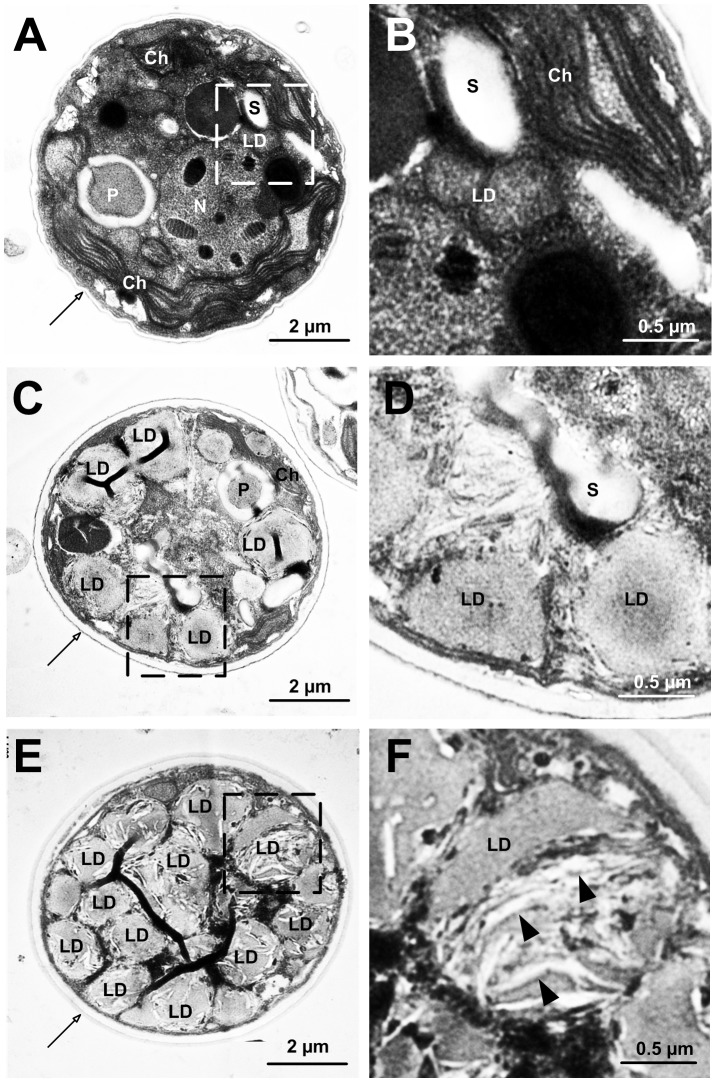
The ultrastructural examination of morphological changes and LD formation in *Symbiodinium* after nitrogen deprivation. Transmission electron micrographs of *Symbiodinium* in control (A, B) and nitrogen-deprivation media (five days: C–D; seven days: E–F). Insets in A, C and D were magnified as B, D and F, respectively. Arrows in A, C, and E indicated cell walls, while arrowheads in F indicated the OsO_4_-negative “inclusion bodies”. Abbreviations: LD, lipid droplet; Ch, chloroplast; S, starch granule; P, pyrenoids; N, nucleolus.

Morphological changes of *Symbiodinium* after nitrogen-deprivation treatment were investigated by TEM (transmission electron microscopy). First, the average cell size (diameter) of *Symbiodinium* cultivated in nitrogen-deprivation medium at day 5 and 7 increased significantly to 7.35±0.86 µm and 6.96±0.96 µm, respectively (*versus* 6.54±1.02 µm in control) (see Table 2). Secondly, the change in cell size was concurrent with cell wall thickness changes (Table 2 and Fig. 4). Specifically, the thickness of the cell wall increased three fold over the controls, from 0.08±0.04 µm to 0.24±0.06 µm at day 5, and then decreased to 0.17±0.05 µm at the 7^th^ day (see also the arrows in Figs. 4A, 4C, and 4E). Thirdly, there were 83.24% and 86.1% reductions in chlorophyll *a* concentration relative to the controls in nitrogen-deprivation cells at day 5 and 7, respectively (Table 2). Finally, there were significant changes in the size and number of LDs after nitrogen-deprivation treatment (Table 2 and Fig. 4). A number of large LDs accumulated in the cytosol of *Symbiodinium* (Figs. 4C and 4E) in nitrogen-deprivation samples. The LDs formation was greatly induced by nitrogen-deprived at day 5, as shown in Figs. 4C-D. Furthermore, the sizes of these LDs significantly increased from 0.72±1.02 µm^2^ to 2.90±1.88 µm^2^ after 7 days of the nitrogen-deprivation treatment (Table 2). Moreover, numerous inclusion bodies, which are OsO_4_ staining-negative, appeared inside the LDs at the 7^th^ day of nitrogen-deprivation treatment (see the arrowhead in Fig. 4F).

**Table 2:** Influence of duration after nitrogen depletion on *Symbiodinium* cells.

	Control	Starvation (Day 5)	Starvation (Day 7)
Cell size (diameter, µm)	6.54±1.02^a^ (n = 86)	7.35±0.86^b^ (n = 123)	6.96±0.96^c^ (n = 110)
Cell wall (thickness, µm)	0.08±0.04^a^ (n = 63)	0.24±0.06^b^ (n = 115)	0.17±0.05^c^ (n = 109)
Chl *a*/protein (µg 100cell^−1^) Reduction of Chl *a* (%)	83.27±0.398 0%	3.98±0.011 95.22%	3.30±0.011 96.03%
LDs size (area, µm^2^)	0.72±1.02^a^ (n = 117)	2.66±1.61^b^ (n = 436)	2.90±1.88^b,c^ (n = 497)

Impact of the nutrient regime in cell pattern between the normal growth and nitrogen starvation (i.e Cell size, LD size, Chl *a*, Cell wall) as analyzed by ANOVA. Upper level a–c denote statistical significance different between control growth, day 5(starvation) and day 7(starvation), respectively (P<0.001). Cell wall and cell size: n =  number of cell analyzed, LD size: n =  number of LDs analyzed.

### Analysis of LD-associated proteins and lipids during the nitrogen-deprivation

**Figure 5:**
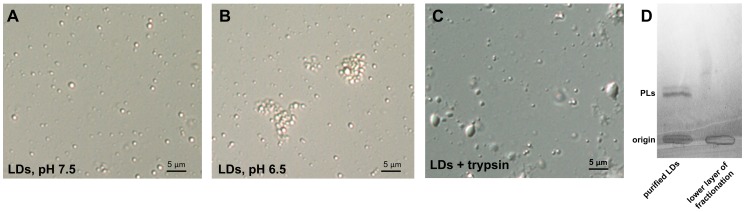
Light microscopy of the LDs purified from *Symbiodinium* cells after different treatments. The LDs were suspended in the (A) pH 7.5 grinding buffer, (B) pH 6.5 grinding buffer or (C) treated by the trypsin digestion. (D) Phospholipid analyses by TLC showing the presence of phospholipids (PLs) in purified LDs (the top layer during the centrifugation) but not lower layer fractions after detergent (0.1% Triton X-100) washing.

LDs were isolated from the cultured *Symbiodinium* incubated in nitrogen-deprivation medium for 5 days. It showed that the LDs purified from the *Symbiodinium* cells maintained as individual particles in a medium of pH 7.5 at 23°C (Fig. 5A). An aggregation of these LDs was induced by lowering the pH of the medium to 6.5; most of these aggregates did not coalesce when left overnight at 23°C (Fig. 5B). After the trypsin treatment, LDs coalesced, floating rapidly and forming a transparent layer on the top of the reaction solution (Fig. 5C). Therefore, the structural integrity of the purified LDs was presumably maintained via the electrostatic repulsion and steric hindrance, in a manner similar to the stable LDs isolated from the *Chlorella* cells [16]. Furthermore, there was significant amount of membrane phospholipids (PLs) in purified LDs but not the lower layer of fractions after the detergent washing (see the “Materials and methods” section) (Fig. 5D). This further indicates that the purified LDs have maintained their membrane integrity during the purification process.

**Figure 6:**
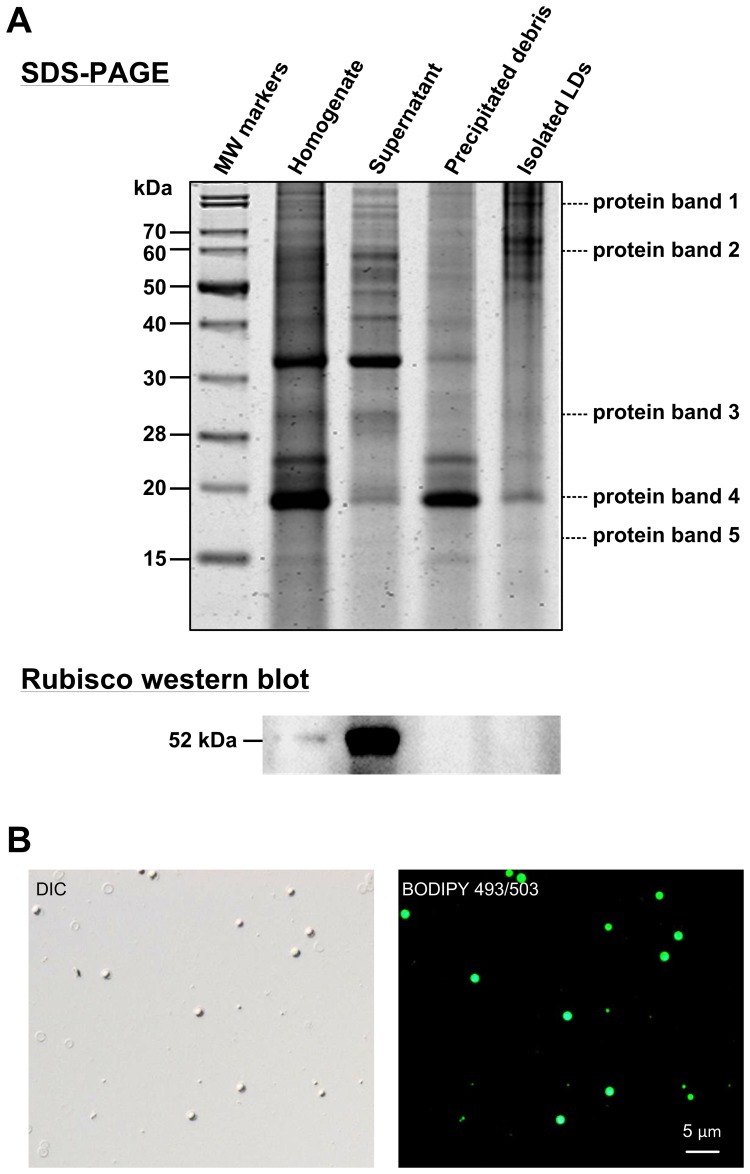
LDs purification and protein analyses. (A) SDS-PAGE analyses of isolated LDs fraction and the LD purity assessment by RuBisCO western blotting. *Symbiodinium* spp. cells harvested after five days of nitrogen deprivation were homogenized and fractionated to purify LDs as shown in the “Materials and methods” section. The purity of LDs was examined based on the absence of RuBisCO contamination by western blotting. Proteins bands 1 to 5 were excised for mass spectrometric analysis. (B) BODIPY 493/503 staining of isolated LDs displayed the abundance of neutral lipid.

The purity of the purified LDs was confirmed based on their absence of a chloroplast-specific protein, RuBisCO, by western blotting according to a published procedure [33] (Fig. 6A). The high lipid contents of the purified LDs were confirmed by strong BODIPY 493/503 staining (Fig. 6B). The TLC analysis showed that the purified LDs contained mainly neutral lipids such as TAGs and CEs (see Fig. 2B), which are both the major lipid component in whole cells as shown in Fig. 2A.

Five major LD-associated protein bands with an estimated molecular weight ranging from 17 to 84 kDa (Fig. 6A) were excised and analyzed by mass spectrometry. Seven proteins were identified, with a variety of cellular functions including lipid metabolism (“Sterol transfer protein” from the protein band 5, MW∼17 kDa), signaling (“14-3-3 protein” from the protein band 3, MW∼28 kDa and “ADP-ribosylation factor (ARF)” from the protein band 4, MW∼19 kDa), stress responses (“Heat shock protein HSP90” from the protein band 1, MW∼84 kDa and “Osmotically inducible protein Y” from the protein band 3, MW∼28 kDa) and energy metabolism (“Mitochondria ATP synthase F1 alpha subunit-like protein 1” from the protein band 2, MW∼59 kDa and “GTP-binding protein” from the protein band 4, MW∼19 kDa) (Table 3).

**Table 3:** Identification of lipid droplet proteins in *Symbiodinium* spp.

Protein name	Species/Taxonomy	Band No.	GI NO.	MS/mps(p)^a)^	Sequence coverage (%)	Predicted MW(kDa)	Observed MW(kDa)	Found with LDs in other organisms	References
**Lipid metabolism**									
Sterol transfer family protein	*Pfiesteria piscicida*/dinoflagellate	5	112253295	72/1(1)	10	11.11	17	Yes	[64]
**Signal-related proteins**									
14-3-3 protein	*Perkinsus marinus*/protozoa	3	294885399	82/3(3)	9	27.39	28	Yes	[65]
ADP-ribosylation factor	*Protozoan*/eukaryotic	4	114131	79/1(1)	5	21.75	19	Yes	[66]
**Stress response**									
Chaperon heat shock protein 90 kDa	*Karlodinium micrum*/dinoflagellate	1	112253669	81/3(2)	5	81.51	84	Yes	[67]
Osmotically inducible protein Y	*Pseudomonas fluorescens*/bacteria	3	229592631	96/1(1)	8	21.02	28	No	[68]
**Energy metabolism**									
Mitochondrial ATP synthase F1 alpha subunit-like protein 1	*Karlodinium micrum*/dinoflagellate	2	319997184	85/3(2)	5	59.51	59	No	[69]
GTP-binding protein	*Helicobacter pylori*/bacteria	4	210134946	73/1(1)	5	19.00	19	Yes	[70]

a)MS/mps(p): Mowse score/number of total matched peptides (numbers of different matched peptides).

## Discussion

### Effects of nitrogen-deprivation on *Symbiodinium* proliferation and morphology

The deprivation of nitrogen, one of the nutrient limitations that critically affect the cellular metabolism, has been shown to induce lipid accumulations in numerous strains of microalgae [13], [34]. *Symbiodinium* spp. is an unique marine microalgae with two living status, either freely living in open ocean or symbiotically residing inside the gastrodermal cells of most marine cnidarian such as sea anemones and corals [1]. Here, we have shown for the first time that free-living *Symbiodinium* accumulate lipids in the form of LDs when the nitrogen source of the culture medium was deprived. Positive growth of *Symbiodinium* spp. was observed after 3 days of the cultivation either under the normal condition or nitrogen starvation. However, it was observed that the slow growth pattern of *Symbiodinium* and accumulation of the LDs occurred under the nitrogen-deprivation (Fig. 1A). This has been reported in several algae that accumulated carbon metabolites under nitrogen-deficient condition, resulting in slow growth and lipid accumulation [35], [36]. However, the nitrogen starvation may also induce an abrupt loss of electron transport capacity in the photosystem II, resulting in the change of the photochemical efficiency (*Fv/Fm*) and growth of *Symbiodinium* [37].

The ultrastructural observation indicated that, after the 7^th^ day of nitrogen deprivation, numerous inclusion bodies started to appear in the LDs (Figs. 4E and 4F). The present study also demonstrated that there was a decreased lipid accumulation in cells on day 9 (Fig. 1B). It was presumed that during day 7 to day 9, *Symbiodinium* used the lipid in order to survive since the reserved nitrogen source had been used up. After 9 days of nitrogen starvation, the *Symbiodinium* cells entered the death phase (Fig. 1A).

Neutral lipids including TAG and CE, are the major lipid species in these LDs. Moreover, concurrently with drastic increases in size and number of LDs, detailed ultrastructural examinations indicated that the increases in cell size and cell wall thickness are typical features induced by the nitrogen deprivation (see Fig. 4 and Table 2). These morphological observations are similar to those reported on the morphological changes in green alga *Scenedesmus obtusiusculus* by nutrient starvation [38], [39]. The increases in cell size and cell wall thickness were probably due to the nutrient deficiency, which led to a delay in cell cytokinesis [40], [41]. Under severe nutrient limitation, cell division-arrested phytoplanktons were found to accumulate glycoproteins and other carbon contents in starch granules. As also shown in the present study, the proliferation of N-deprived *Symbiodinium* decreased significantly in comparison to the cells grown in normal medium (Fig. 1A). Similar inhibitory effects of nitrogen limitation on the proliferation and morphology of *Symbiodinium* in hospite have also been reported previously in two hermatypic corals (*Porites porites* and *Montastrea annularis*) and a sea anemone (*Aiptasia pallida*) [21], [42].

### The Carbon/Nitrogen (C/N) relocation during the nitrogen-deprivation and its implication for the endosymbiotic regulation

The decreased proliferation rates in most microalgae due to nitrogen deprivation have been reported to be concurrent with increases of total lipid levels [43], [44]. This was also confirmed by the present study, indicating that there is a reduced synthesis of new membrane constituents, in the nitrogen-deprived *Symbiodinium*. Here, the cell machinery shifted its metabolism to synthesize higher levels of TAGs and CEs for storage in LDs (Figs. 2–4).

The depletion of nitrogen could break the balance of carbon (C) and nitrogen (N) availability, switching the cellular metabolism toward the synthesis of carbon-containing compounds (i.e. lipids and/or carbohydrates); which then eventually leads to a higher cellular C:N ratio [45], [46]. For example, the increase of lipid in microalgae upon nitrogen-deficiency could result from the partition of the excess carbon to intracellular lipid storage [47]. As shown in the present study, under nitrogen-deprived environment of free-living *Symbiodinium*, excess carbon allocates to the lipid pool and results in lipid accumulation as a form of LDs. Other environmental stresses, such as irradiance and various ecological changes, could also impact the C:N allocation in marine diatoms [48], [49].

Besides amino acids, ammonium and nitrate have been shown to be sources of nitrogen in cultured and symbiotic *Symbiodinium* [6], [50]. The investigation using the synchrotron radiation spectroscopy has further confirmed that symbiotic *Symbiodinium* (i.e. *in hospite*) contained less nitrogen compounds and more lipids than free-living *Symbiodinium* [9]. It has indicated the cytosol of the gastrodermal cell could be a nitrogen-limited environment, by which the symbiotic association with endosymbionts is regulated [51]. It is feasible that, by a mechanism of nitrogen limitation, cnidarian hosts regulate their symbiotic *Symbiodinium* to elevate lipid contents and store in a form of LDs [5], [8], [9].

### The nitrogen-deprivation induced quantitative and qualitative changes of lipids in *Symbiodinium*

The most abundant fatty acid components in TAGs of *Symbiodinium* cultivated in the standard f/2 media (Fig. 3) were found to be myristic acid (C14), palmitic acid (C16), stearic acid (C18) and erucic (22∶1), representing a fatty acid composition similar to that of higher plants [52]. These fatty acids are also common in other algae [19]. Palmitic acid (C16) and docosahexaenoic acid (C22:6) were the most abundant fatty acids in TAGs of nitrogen-deprived *Symbiodinium* cells. It has been proposed that TAG biosynthesis in microalgae may consist of three steps: (1) formation of acetyl coenzyme A in the cytoplasm, (2) elongation and desaturation of the carbon chain of fatty acids in the chloroplast, and (3) synthesis of TAG in the endoplasmic reticulum [53]. In the second step, C16 and C18 fatty acids are formed prior to the production of other long-chain fatty acids or PUFAs, which requires further elongation and desaturation. Therefore, the change of C16:0 and C18:0 levels in nitrogen-deprived *Symbiodinium* documented herein may imply the accelerated synthesis and accumulation of PUFAs in the LDs, such as C16:1, C18:1, C18:2, C20:5,C21:5, C22:1, and C22:6. PUFAs are of the utmost importance for human metabolism [54]. For example, the long-chain docosahexaenoic acid (DHA) provides significant health benefits to the human population, particularly in preventing cardiac diseases such as arrhythmia, stroke and high blood pressure [55], [56]. Accordingly, an increase in the PUFA production by microalgae has been shown to be important for many nutritional and pharmaceutical purposes [57].

### The proteomics of LDs

Using the neutral lipid probe BODIPY 493/503, we were able to visualize the LDs isolated from cultured *Symbiodinium*, with 0.5–1 µm in diameter (Fig. 6B). Oil bodies of similar sizes (0.5–2 µm diameter) have previously been reported in plant seeds [15]. However, the protein compositions of LDs between the *Symbiodinium* and other plant seeds are obviously different, indicating distinct regulatory mechanisms [58]. For example, other LD-related proteins, such as oleosin, have been discovered to be abundant in microalgae and plant seeds [16], [19], [59]. Both N- and C- terminal domains of oleosins have been proposed to reside on the LD (or so called “Oil Bodies”) surface to stabilize this organelle via steric hindrance and electronegative repulsion [60]. Nevertheless, oleosins were not identified in the LDs of *Symbiodinium* spp. as shown in this study, demonstrating a different formation mechanism during the endosymbiosis. As a consequence, the examination of protein compositions of LDs in nitrogen-deprived free-living *Symbiodinium* should provide new insights to elucidate the LD formation mechanism. Besides proteins responsible for lipid and energy metabolisms, other LD-associated proteins involving in signaling (14-3-3 protein and ARF) and stress response (HSP90 and the osmotically inducible protein Y) were also identified (see Table 3). The 14-3-3 proteins are a ubiquitous group of signaling proteins that involve in many regulatory functions [61] such as carbohydrate and lipid metabolisms in plants [62]. Furthermore, both ARF and GTP-binding proteins are key regulators in membrane trafficking, and thus play pivotal role in LDs biogenesis [63].
